# Evaluating the Effectiveness and Safety of Robotic-Assisted MRI/TRUS Fusion Transperineal Prostate Biopsy Systems: A Narrative Review Based on Current Literature

**DOI:** 10.5152/eurasianjmed.2023.23370

**Published:** 2023-12-01

**Authors:** Akif Erbin, Ufuk Caglar, Rustu Turkay

**Affiliations:** 1 Department of Urology, Haseki Training and Research Hospital, Istanbul, Turkey; 2 Department of Radiology, Haseki Training and Research Hospital, Istanbul, Turkey

**Keywords:** Biopsy, magnetic resonance imaging, prostatic neoplasms, robotics

## Abstract

Robotic-assisted magnetic resonance imaging (MRI)/transrectal ultrasound (TRUS) fusion transperineal biopsy systems are one of the most debated and interesting subjects both in practice and in current urology literature. The comprehensive literature research was carried out in the PubMed/MEDLINE, Google Scholar, and Scopus databases using the terms “robotic transperineal prostate biopsy,” “robot-assisted MRI/US fusion biopsy,” “robot-assisted MRI/TRUS fusion biopsy,” or “robotic targeted prostate biopsies.” All article types were included in the study (n = 343). Among these, articles in non-English languages, duplicate articles, review articles, guidelines, and book chapters were excluded from the study (n = 325). Additionally, articles on In-bore biopsy and semirigid device techniques were also excluded from the study (n = 5). A total of 13 original research studies (3 retrospective and 10 prospective nonrandomized studies; total number of patients = 1844) performed with 2 different robotic-assisted transperineal biopsy platforms (iSR’obot™ MonaLisa, Biobot Surgical, Singapore; and Artemis™, Eigen,GRASS VALLEY, USA) were analyzed in detail. The overall cancer detection rates ranged from 51.2% to 73.7%, while the rates of detecting clinically significant (cs) prostate cancer (Pca) ranged from 23.0% to 52.7% in patients who had not been previously diagnosed with prostate cancer. Among the 1844 patients, only 2 individuals (0.01%) were diagnosed with urosepsis. Although the role of these devices in prostate biopsies is not completely clear, the robot-assisted transperineal prostate biopsy technique is an effective and safe procedure, with high rates of csPCa detection and acceptable rates of complications, especially in terms of urosepsis.

## Introduction

A prostate biopsy is still an indispensable method for diagnosing prostate cancer (PCa).^1^ Prostate biopsy, one of the most frequently performed urological procedures, is performed in Europe and the United States of America over 1 million times a year.^2^ Overdiagnosis of clinically insignificant (cis) PCa in prostate biopsies guided by transrectal ultrasound (TRUS) has created the need for advanced diagnostic methods.^3^ Hence, in recent times, advanced approaches utilizing multiparametric magnetic resonance imaging (Mp-MRI) have been prominent in the field of diagnosing PCa. So far, 3 Mp-MRI guided prostate biopsy techniques have been introduced, including MRI/TRUS fusion biopsy, cognitive targeted biopsy (COG-TB), and in-bore biopsy performed directly under MRI guidance without fusion. These techniques increased the rates of clinically significant (cs) PCa diagnoses and reduced cisPCa rates.^4^ Thus, the risk of overdiagnosis and overtreatment has been reduced.

Transperineal prostate biopsies have gained more popularity in recent years because of their greatly reduced risk of infection compared to the transrectal route. This approach is particularly apparent in prostate fusion biopsies (MRI/TRUS fusion biopsy and COG-TB). Together with new platforms developed, MRI/TRUS fusion tranperineal biopsy has become more widely used in urology practice.^5^ Robotic systems, which are useful for diagnosing and treating many diseases, have also been integrated into the MRI/TRUS fusion biopsy platforms to improve the success of biopsies and lower the number of mistakes made by humans. Thanks to these developments in technology, many new robotic-assisted MRI/TRUS fusion transperineal biopsy systems have been released in the last few years, and many studies have been published about them.

Robotic-assisted MRI/TRUS fusion transperineal biopsy systems are one of the most debated and interesting subjects in the field of urology, both in practice and in current urology literature. The aim of this narrative review study is to analyze the findings of original research articles that focused on evaluating the efficacy and safety of MRI/TRUS fusion transperineal prostate biopsy systems.

## Materials and Methods

**Figure 1.**
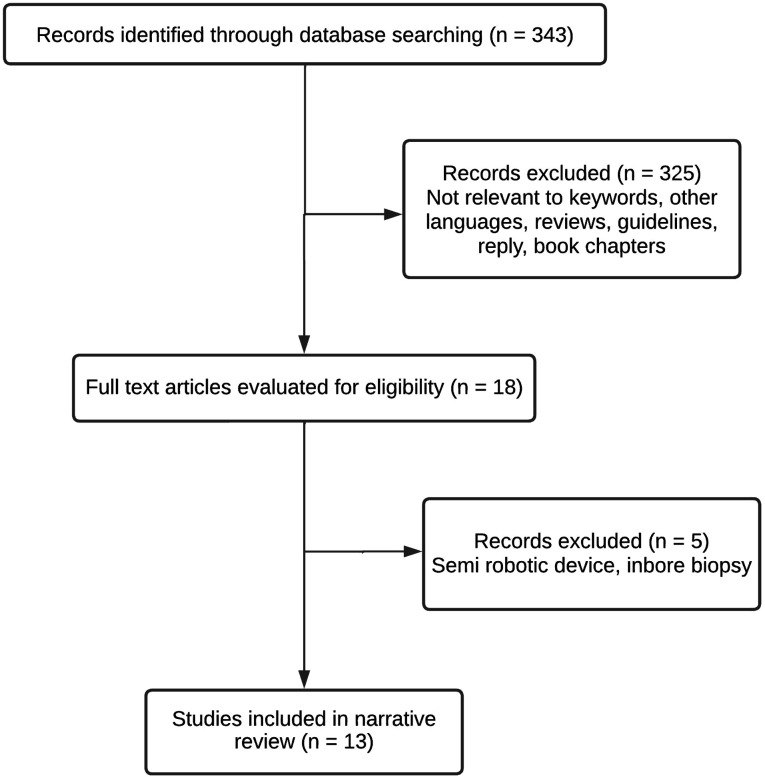
Flowchart of the study.

A comprehensive literature study was conducted to identify studies related to robotic-assisted MRI/TRUS fusion transperineal prostate biopsy. The comprehensive literature research was carried out in the PubMed/MEDLINE, Google Scholar, and Scopus databases using the terms “robot-assisted MRI-US fusion biopsy,” “robotic targeted prostate biopsies,” or “robotic transperineal prostate biopsy.” Several supplementary derivatives of these fundamental phrases were also used to enhance the search and efficiently scan the whole body of literature. All identified article types were included in the study. Among these, articles in non-English languages, duplicate articles (articles in both databases), review articles, guidelines, and book chapters, articles containing semirigid device techniques (devices that are not fully compatible with the robotic assisted system), irrelevant articles after a term-based search, and articles related to in-bore biopsy were excluded from the study. Finally, a total of 13 original research papers (3 retrospective and 10 prospective nonrandomized studies) were analyzed in this presented narrative review study that fully met the criteria (Figure 1). Articles included in the study contained publications that were meticulously chosen for publication in English, had a quantitative design, and made original scientific contributions.

### MRI/TRUS Fusion Transperineal and Transrectal Prostate Biopsy

An MRI/TRUS prostate fusion biopsy can be performed both transrectally and transperineally. Both methods have their own advantages and disadvantages. Due to its association with the rectal mucosa, transrectal biopsy carries a high risk of infection.^6^ Therefore, the European Urology Association prostate cancer guidelines recommend that the biopsy of the prostate be performed primarily by the transperineal approach.^7^ Although transperineal prostate biopsy is advantageous for infectious complications, it is associated with drawbacks such as a long learning curve and a longer procedural duration when compared to transrectal biopsies.^8^ There are also disadvantages, such as difficulty targeting lesions in very small prostates and the impossibility of needle passage in large prostates that are situated beyond the bone-pelvic window. Despite all these disadvantages, the transperineal method should be preferred, if possible, because the risk of fatal complications such as acute prostatitis and sepsis is much lower. While there has been a partial increase in the utilization of the transperineal approach in recent years, the transrectal procedure remains significantly more favored among urologists. The main reason for this is that the transrectal pathway has been used for a much longer time, is seen as a simpler method, and presents a shorter distance to reach the prostate, unlike the transperinel pathway. One of the most important advantages of transrectal biopsy is that it can be performed in office conditions under local anesthesia. Due to the availability of these advantages, the majority of prostate biopsies in the United States continue to be conducted by the transrectal method.^9^ However, recent studies have shown that a transperineal prostate biopsy can also be performed under local anesthesia.^10^ A group of scientific researchers emphasized that transrectal biopsy should be abandoned and switched to transperineal biopsy due to the risks of not only infection and death but also financial burden. In 2020, they initiated the ‘‘TRexit’’ movement and advocated for a carefully organized worldwide withdrawal from the transrectal approach, with a gradual elimination of transrectal biopsy led by proficient transperineal biopsy centers, and they proposed that this process should be completed by the end of 2022.^11^ Nevertheless, the “TRexit” movement is currently not progressing according to initial expectations as we near the year 2024.

### Robotic-A ssisted MRI/TRUS Fusion Transperineal Prostate Biopsy Systems

Robot-assisted biopsy equipment is equipped with a robotic arm that autonomously regulates the position of the biopsy, the angle of the needle, and the depth of the procedure. They facilitate the accurate, 3-dimensional targeting of the biopsy needle toward the specific lesion. 

**Figure 2.**
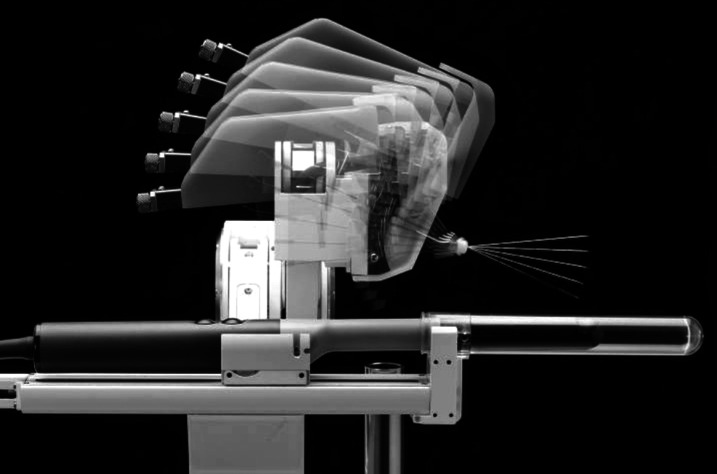
iSR’obot™ MonaLisa robotic-assisted MRI/TRUS fusion transperineal prostate biopsy system with transrectal ultrasound probe (BK 3000; BK Medical, Peabody, Mass, USA).

iSR’obot™ MonaLisa (Biobot Surgical Ltd., Singapore) robotic-assisted biopsy platform is an up-to-date automation system that provides prostate biopsy to be taken via a transperineal approach (Figure 2).^12^

The iSR’obot™ MonaLisa robotic-assisted transperineal biopsy system creates a map of the prostate gland and existing lesions using preprocedure Mp-MRI images, and real-time TRUS images are then focused on this map during the procedure.^13^ This approach can be considered a minimally invasive technique, as it allows for complete access to the prostate through 2 perineal holes. Therefore, it provides low complication rates and high patient comfort. It is claimed that this system minimizes neurovascular bundle damage.^14^

**Figure 3.**
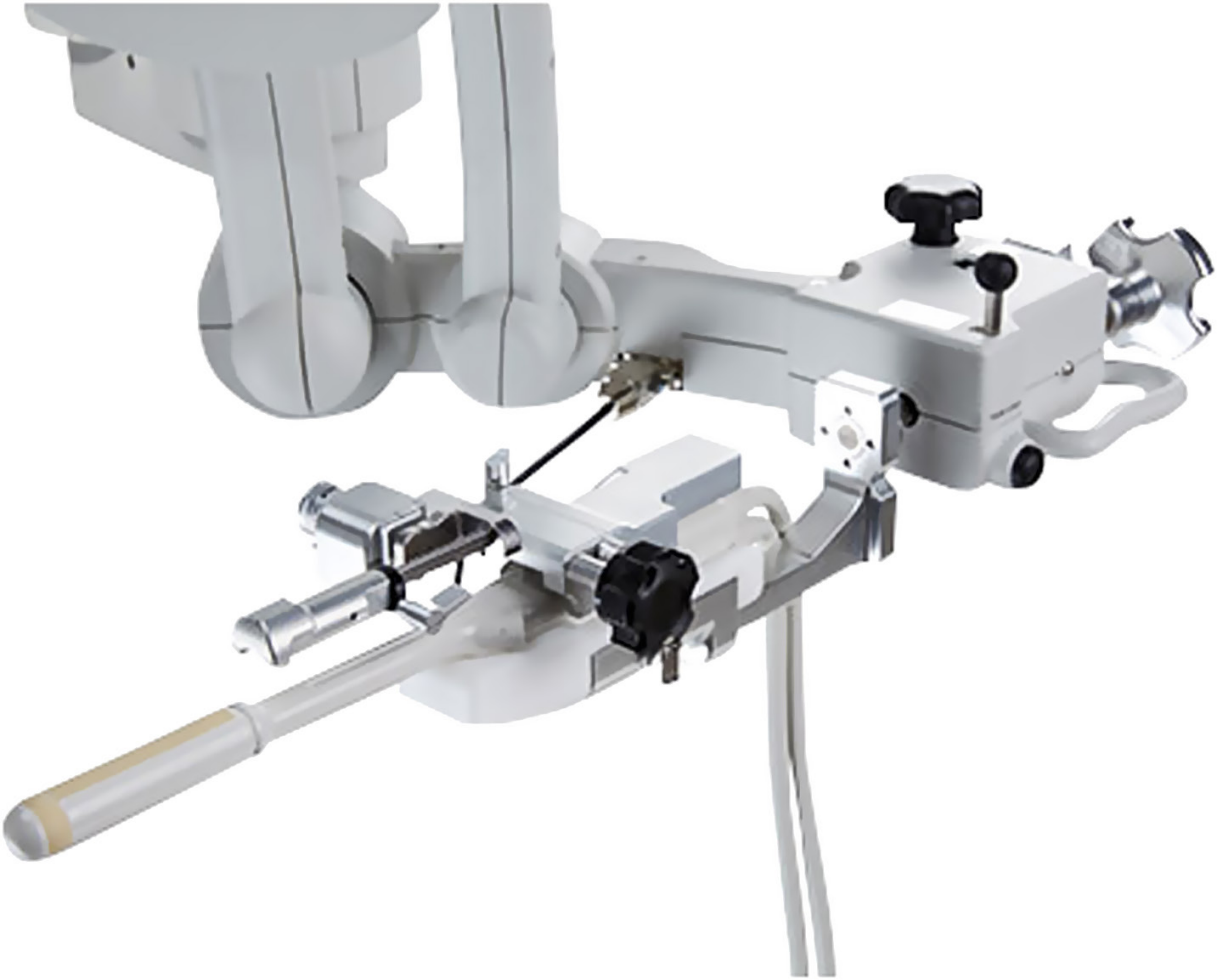
Artemis™ robotic-assisted MRI/TRUS fusion prostate transperineal biopsy platform.

Artemis is a multiparameter MRI/US technology that can precisely guide needles to identify lesions and improve the accuracy of biopsies by utilizing the robotic fusion biopsy system and robotic electronic-mechanical tracking of the prostate. The system collects 360° ultrasound images and uses them to construct a 3D virtual model of the prostate. It also generates a computer-generated outline of the prostate, which may be further adjusted by the doctor. By integrating software that generates 3-dimensional models of the prostate using MRI, the ARTEMIS device is capable of mapping suspicious lesions. Subsequently, this radiological data can be aligned and combined with the ultrasound model (Figure 3).

While research has demonstrated the efficacy and safety of robot-assisted transperineal prostate biopsy, the specific function of these devices in transperineal prostate biopsies remains uncertain.

### Results

#### Cancer Detection Rates of Robotic-Assisted Transperineal Fusion Prostate Biopsy

The growing utilization of Mp-MRI in regular clinical practice has resulted in substantial decreases in the overdiagnosis of cisPca. Seung et al demonstrated that patients who underwent Mp-MRI follow-up experienced a reduction in hospital admissions and incurred reduced costs for their follow-up compared to those who underwent TRUS biopsies.^15^ It is essential not to miss the clinical diagnosis of csPca as well as to reduce the unnecessary diagnosis of cisPca. The incorporation of robotic devices into Mp-MRI prostate biopsy has great promise in reducing error margins and increasing csPca rates.

Cancer detection rates of robot-assisted transperineal fusion prostate biopsy were evaluated in 11 of the 13 studies included in this review study (Table 1). When examining patients who had not been previously diagnosed with prostate cancer, the overall rates of detecting cancer ranged from 51.2% to 73.7%, while the rates of detecting csPca ranged from 23.0% to 52.7%. Chen et al evaluated data only from patients who were followed up with active surveillance. The study conducted on a sample of 19 patients revealed an overall cancer detection rate of 73.7%, with a csPca detection rate of 15.8%.^16^ Lee et al demonstrated that robotic-assisted transperineal target biopsy outperformed systemic biopsy in terms of both overall and csPca rates (*P* < .001, *P* < .001, respectively).^17^ Patel et al compared the results of robot-assisted transperineal biopsy with cognitive targeted biopsy (COG-TB) performed without robot assistance and reported that CsPca detection rates were significantly higher in the robot-assisted transperineal biopsy group (* P* = .014).^18^ Claros et al compared the results of COG-TB performed with micro-ultrasonographyand robot-assisted transperineal biopsy. Their findings demonstrated that the micro-ultrasound biopsy yielded considerably higher rates of CsPca (*P* = 0.002).^19^ In their prospective study, Kauffmann et al compared 3 different biopsy techniques and reported that robot-assisted transperineal biopsy had significantly higher cancer rates than COG-TB and in-bore biopsy techniques (* P* = .002).^20^

#### Safety Profile of Robotic-Assisted Fusion Transperineal Prostate Biopsy Systems

Although prostate biopsy is a minimally invasive procedure, there is a possibility of experiencing complications either during or after the procedure. The possible complications are hematuria, rectal hemorrhage, hematospermia, acute urine retention, perineal hematoma, acute prostatitis, and the gravest complication, sepsis.^21^ Multiple investigations have demonstrated that the occurrence of acute prostatitis and urosepsis following a transperineal prostate biopsy is extremely low.^22,23^ The transperineal approach offers a clear benefit over the transrectal technique in terms of greatly reducing the occurrence of infective complications. In 5 of the studies we included in the review, they focused only on cancer detection rates, while the safety of prostate biopsy was not assessed. Miah et al analyzed the data of 86 individuals and reported that just 1 patient experienced urosepsis, which necessitated hospitalization.^12^ In their investigation, Walter et al observed a rate of 0.4% for urinary tract infections that did not necessitate hospitalization.^24^ Yang et al reported that urosepsis developed in 1 out of 30 patients.^25^ No instances of urosepsis complications were detected in other studies (Table 1).

The most common complications of prostate biopsy are hematuria and hematospermia.^26^ In their investigation on the safety of transperineal robotic biopsy, Walter et al documented a hematospermia rate of 43%. The researchers assessed the incidence of hematuria based on the duration of occurrence and found that the rates for hematuria lasting 1 day, 2-3 days, and more than 3 days were reported as 14.9%, 22.4%, and 25.9%, respectively.^24^ The incidence of minor hematuria ranged from 7.5% to 9.0%, while the incidence of severe hematuria ranged from 1.8% to 1.9% in other investigations.^18,20,27^

In addition to bleeding and infectious problems, another bothersome symptom is acute urine retention (AUR). The incidence of AUR varied between 1.9% and 5.4% among the studies evaluated.^18,28^ Walter et al evaluated the risk factors associated with the occurrence of an AUR following a robot-assisted transperineal prostate biopsy and revealed that having a high number of biopsy cores (≥ 25) and a large prostate volume (≥40 mL) were significant risk factors for the development of an AUR.^24^

In the sole study assessing the outcomes of robot-assisted transperineal prostate biopsy in relation to patient comfort, it was demonstrated that there was no difference in terms of international prostate symptom score, the international consultation on incontinence questionnaire, or the quality of life values of 228 patients prior to and following the procedure.^24^ Furthermore, the study also assessed the visual analog scores (VAS) of the patients, but no significant change was detected.^24^

Trotsenko et al evaluated the relationship between robot-assisted transperineal prostate biopsy and erectile dysfunction and found no significant difference in terms of international index of erectile function scores before and after the procedure. In addition, subgroup analyses (age, number of biopsy cores, previous biopsies) were performed in the study, and it was reported that there was no relationship between prostate biopsy and erectile dysfunction for each subgroup.^14^

### Discussion

#### Anaesthetic Techniques for Robotic-Assisted Fusion Transperineal Prostate Biopsy

A prostate biopsy can be performed using several methods of anesthesia, including local anesthesia, periprostatic blocking, pudendal nerve blockade, sedoanalgesia, caudal block, spinal anesthesia, and general anesthesia. It is thought that transrectal biopsy requires less anesthesia than transperineal biopsy.^29^ Recent studies have shown that a transperineal prostate biopsy can be performed without the need for sedoanalgesia.^30^ Some authors argue that the use of general anesthesia will enhance the success of the treatment by minimizing patient movement.^31^

Out of the 13 studies we examined in this study, only 1 of them included a comparison of anesthetic applications. Walter et al demonstrated that there was no statistically significant difference in VAS score during a robot-assisted transperineal prostate biopsy between patients who underwent the procedure with local anesthesia and those who received it with general anesthesia.^24^ However, no different evaluation was made in the study in terms of success with the anesthesia technique, surgeon comfort, or complications. Among the remaining 12 publications, anesthetic technique information was absent in 4 articles, while in the other 8 articles, it was explicitly mentioned that the procedure was conducted under general anesthesia for all patients.

The optimal anesthetic strategy for robot-assisted transperineal prostate biopsy has not been clear yet due to a lack of consensus and the absence of prospective randomized controlled trials (RCTs). It is foreseeable that the implementation of the robotic technology will decrease the requirement for anesthesia due to its ability to perform the procedure using 2 perineal accesses and accurately target the lesion.

#### Antibiotic Prophylaxis in a Robotic-Assisted Fusion Transperineal Prostate Biopsy

Many urologists routinely administer prophylactic antibiotics to prevent urosepsis, the most severe complication that can occur following a prostate biopsy. Despite the very low incidence of urosepsis in transperineal prostate biopsy, routine antibiotic prophylaxis was used before transperineal biopsy in 98 of the 106 studies reviewed in a recent meta-analysis.^32^

Not a single publication included in the study specifically addressed the efficacy and necessity of antibiotic prophylaxis. Walter et al administered antibiotic prophylaxis to 33% of the patients. Wetterrauer et al reported that antibiotic prophylaxis was given to 50% of the patients. Patel et al and Mischinger et al both indicated that antibiotic prophylaxis was administered to all patients.^18,^^24,33,34^ However, these articles do not contain detailed information about the antibiotic prophylaxis applied. Miah et al administered a single dose of IV gentamicin to patients,^12^ while Yang et al aimed to provide prophylaxis with oral amoxicillin clavulonate.^25^

Currently, there is no clear recommendation on whether antibiotic prophylaxis is necessary prior to robot-assisted transperineal prostate biopsy or transperineal prostate biopsy in general. In a recent meta-analysis conducted by Castellani et al, consisting of 4 retrospective studies and 4 prospective and nonrandomized studies, it was found that administering antibiotic prophylaxis before a nonrobotic transperineal prostate biopsy had no impact on fever, sepsis, or hospitalization.^35^ The publication of RCTs in the future will provide a definitive understanding of the requirement for antibiotic prophylaxis before a transperineal prostate biopsy.

Although the role of these devices in prostate biopsies is not completely clear, robot-assisted transperineal prostate biopsy systems are an effective and safe procedure with high rates of csPCa detection and acceptable rates of complications, especially in terms of urosepsis. In order to determine the place of the robotic-assisted MRI/TRUS fusion transperineal prostate biopsy approach in clinical practice, it is necessary to carry out RCTs that directly compare it with previous biopsy systems.

#### Limitations

This article presents a comprehensive current literature review on the subject of robotic-assisted transperineal prostate biopsy and assesses the outcomes achieved through this technique. However, there are some limitations to the study. Despite our comprehensive search across many databases, it is possible that certain pertinent studies may have been overlooked. Furthermore, this study was carried out in accordance with the data sets and resources utilized, and its scope is limited by the limits inherent in these resources. Due to the review’s focus on present technical advancements, it may present challenges to forecasting the potential effects of future breakthroughs and innovations. Finally, due to the absence of any research examining the expenses associated with robotic prostate biopsy devices, this analysis is unable to provide specific information regarding their cost. Despite all these limitations, this specific review study will contribute to the literature and clinical practice by summarizing the results of robotic-assisted MRI/TRUS fusion transperineal prostate technique that can be considered new. 

## Figures and Tables

**Table 1.** Characteristics and Results of the Reviewed Articles (n = 13)

iSR’obot™ MonaLisa (Biobot Surgical, Singapore)					
Author, Year	Type of Study	N	Design of the Study	Cancer Detection Rate	Complication Rate
Trotsenko et al 2023^14^	Prospective	157	Primary results	–	–
Walter et al 2022^24^	Prospective	228	Primary results	–	14%
Wetterauer et al 2021^33^	Prospective	118	Systematic Versus Targeted Biopsy	1 core: 39.1% 2 cores: 52.2% 3 cores: 67.4%	–
Yang et al 2020^25^	Prospective	30	Primary results	63.3% (all) 50.0% (csPca)	6.7% 1 urosepsis 1 Aur
Lee et al 2020^17^	Retrospective	433	Systematic Versus Targeted Biopsy	%57 %46 (csPca)	–
Patel et al 2020^18^	Retrospective, multicentre	92	COG-TB Versus Targeted Biopsy	60.4% (all) 39.6% (csPca)	Aur 1.9% Haematuria 7.5% Perineal bruising 8%
Miah et al 2019^12^	Prospective	86	Primary results	51.2% (all)	1 urosepsis
Mischinger et al 2018^34^	Prospective	232	Primary results	61% (all) 45.3% (csPca)	3% 1 rectal injury/peritonitis 6 Aur 3 Perineal bruising
Kaufmann et al 2018^20^	Prospective	73	Robotic TP, In‐bore and COG-TB	52.4% (all) 35.6% (csPca)	–
Chen et al 2017^16^	Prospective	19	Combination MRI-targeted and transperineal template biopsy	73.7% (all) 15.8% (csPca)	–
Kaufmann et al 2017^28^	Prospective	55	Primary results	61.8 (all) 52.7% (csPca)	Aur 5.4% Major bleeding 1.8% Minor bleeding 9.1%
Kroenig et al 2016^27^	Retrospective	52	Primary results	59.6% (all) 51.9% (csPca)	2 patients (temporary bleeding and a rectum perforation)
**Artemis™ (Eigen, USA)**					
**Author, Year**	**Type of Study**	**N**	**Design of the Study**	**Cancer Detection Rate **	**Complication Rate**
Claros et al 2020^19^	Prospective	269	Cognitive microUSG Versus Robotic Tp	23% (csPca)	–

AUR, acute urinary retention; COG-TB, cognitive targeted biopsy; csPca, clinically significant prostate cancer; MRI, magnetic resonance imaging; TP, transperineal; USG, ultrasonography.
